# Draft genome sequence of *Bacillus* sp. EE-W1 isolated from a biogas reactor

**DOI:** 10.1186/s13104-020-04991-w

**Published:** 2020-03-14

**Authors:** Waleed S. Mohammed, Elvira E. Ziganshina, Ramilya F. Akhmetova, Ayrat M. Ziganshin

**Affiliations:** 1 Department of Biotechnology, Faculty of Agriculture, Al-Azhar University, Cairo, 11651 Egypt; 2 Department of Microbiology, Kazan (Volga Region) Federal University, Kremlyovskaya str. 18, Kazan, 420008 Russia

**Keywords:** Draft genome, *Firmicutes*, *Bacillus*, Biogas reactor, Illumina sequencing

## Abstract

**Objectives:**

The genus *Bacillus* comprises spore-forming rod-shaped Gram-positive bacteria, which usually grow aerobically or anaerobically. Members of this genus are common environmental microorganisms. Also, they can be monitored in the food production chain. Genome sequence of *Bacillus* sp. strain EE-W1 will provide helpful information to understand its ecology and genetics. Draft genome data may be useful in the field of using *Bacillus* species in industrial biotechnology. Also, these data can be a useful resource for the study of comparative genomics.

**Data description:**

Here, we present the draft genome sequence of *Bacillus* sp. strain EE-W1 isolated from a biogas reactor, Kazan, Russia. The assembled genome size was 5,769,164 bp, with a GC content 35.1%. This draft genome data can be accessed at DDBJ/ENA/GenBank under the accession WIPE00000000.

## Objective

The genus *Bacillus* includes spore-forming rod-shaped Gram-positive aerobic or facultative anaerobic bacteria. A distinctive feature of the genus *Bacillus* is its ability to form endospores in response to various environmental and nutritional stresses. Members of this genus are common environmental microorganisms. Also, they can be monitored in the food production chain [1]. Moreover, some species of *Bacillus* with high proteolytic activity have demonstrated a sustained survival in some protein-fed anaerobic digesters, what finally led to an improved biogas productivity [2]. However, the *Bacillus* group also includes pathogenic species, therefore, it is necessary to develop proper hygiene and sanitation procedures to decrease the potential risk of transmission of the infectious disease [3]. Genome sequence of *Bacillus* sp. strain EE-W1 will provide helpful information to understand its ecology and genetics. Draft genome data can be also useful in the field of using *Bacillus* species in industrial biotechnology. Also, these data can be a useful resource for the study of comparative genomics.

## Data description

The *Bacillus* sp. EE-W1 was isolated from a laboratory-scale mesophilic biogas reactor. Briefly, 1 g (wet weight) of digested wastes was consistently diluted with sterile tap water. 100 µL of each obtained dilution was spread onto LB agar plates and then cultured at + 37 °C. Finally, single colonies were transferred to new plates, and the cultivation process was repeated until pure cultures were received. For DNA extraction, the bacterial strain *Bacillus* sp. EE-W1 was cultured on LB agar at + 37 °C for 2 days. Genomic DNA from the *Bacillus* sp. EE-W1 was then extracted using a commercially available DNA extraction kit, FastDNA spin kit (#116540600; MP Biomedicals, USA), and assessed by spectrophotometric analysis as reported previously [4, 5]. The traditional identification of the strain EE-W1 was carried out using morphological characteristics and biochemical tests followed by the PCR-amplified 16S rRNA gene sequence analysis (Table 1) at the EzBioCloud Database [6]. The phylogenetic tree was then constructed with the MEGA 7 software [7]. The genome sequence of *Bacillus* sp. EE-W1 was received by Illumina sequencing as described previously [5]. Sequence read quality was checked using FastQC v0.11.5 [8] and trimmed with Trimmomatic version 0.36 [9]. The filtered reads were assembled into contigs with minimum contig length 500 bp using de novo assembler Velvet version 1.2.10 [10]. Contigs were ordered using progressive Mauve version 2.4.0 [11]. The quality of genome assembly was estimated with Quast version 5.0.0 [12]. Gene prediction and annotation was performed using the Rapid Annotation System Technology (RAST) server [13]. The rRNA genes were detected by using Barrnap version 0.9 [14], while tRNA genes were identified with Aragorn version 1.2 [15]. The phylogeny based on the whole genome data (Table 1) was determined by calculating the average nucleotide identity with closely related *Bacillus* species with the Orthologous Average Nucleotide Identity Tool (OrthoANI) [16].

**Table 1:** Overview of data files/data sets

Label	Name of data file/data set	File types (file extension)	Data repository and identifier (DOI or accession number)
Bacillus sp. EE-W1, whole genome shotgun sequencing project	Whole genome shotgun project	FASTA	Genbank (https://identifiers.org/ncbi/insdc:WIPE00000000.1)
Bacillus sp. (in: Bacteria) strain EE-W1 16S ribosomal RNA gene, partial sequence	16S rRNA gene sequence	FASTA	Genbank (https://identifiers.org/ncbi/insdc:MN611309.1)

The calculated OrthoANI values with closely related species showed that the strain EE-W1 presented OrthoANI values of 96.33% and 94.79% with *Bacillus wiedmannii* strain FSL W8-0169 and *Bacillus mobilis* strain 0711P9-1 (type strains), respectively. The draft genome assembly of *Bacillus* sp. EE-W1 consisted of 169 contigs larger than 500 bp, with a total size of 5,769,164 bp and mean GC content 35.1%. The obtained genome sequence data was at 117× coverage. The RAST server predicted 6104 coding sequences. The genome of *Bacillus* sp. EE-W1 encodes at least 3 rRNAs and 8 tRNAs. The genome of the strain EE-W1 also harbored genes related to proteins and carbohydrates degradation and fermentation (including mixed acid, lactate fermentation, acetyl-CoA fermentation to butyrate, acetoin, butanediol metabolism, and butanol biosynthesis). In addition, the genome contained various genes related to resistance to several toxic compounds and some antibiotics (such as streptothricin, tetracycline and fosfomycin).

## Limitations

Current data is based on the draft level genome sequence, due to which exact length of the genome, synteny, number of rRNA and repetitive elements cannot be certainly reported.

## Data Availability

The data described in this Data note can be freely and openly accessed at DDBJ/ENA/GenBank. Accession Numbers—https://identifiers.org/ncbi/insdc:WIPE00000000.1 (whole genome project) [17] and https://identifiers.org/ncbi/insdc:MN611309.1 (16S rRNA gene sequence) [18].
